# Aproximación ecológica a las características y factores determinantes de la violencia sexual contra adolescentes en Perú

**DOI:** 10.7705/biomedica.7131

**Published:** 2024-05-30

**Authors:** Yordanis Enríquez, Claudia Rebeca Cahui, Giovani Martín Díaz

**Affiliations:** 1 Facultad de Ciencias de la Salud, Universidad Católica Sedes Sapientiae, Lima, Perú Pontificia Universidad Católica del Perú Facultad de Ciencias de la Salud Universidad Católica Lima Peru

**Keywords:** delitos sexuales, prevalencia, exposición a la violencia, adolescente, factores de riesgo, modelos teóricos, Perú, sex offences, prevalence, exposure to violence, adolescent, risk factors, models, theoretical, Perú

## Abstract

**Introducción.:**

La violencia sexual contra los adolescentes es un problema global que afecta a jóvenes de todo el mundo. El modelo ecológico examina sus formas y factores determinantes a través de niveles interconectados.

**Objetivo.:**

Determinar la frecuencia, las características y los predictores de la violencia sexual contra adolescentes escolarizados en Perú.

**Materiales y métodos.:**

Se llevó a cabo un estudio transversal en el cual se analizaron de manera secundaria los datos de la Encuesta Nacional de Relaciones Sociales (2019). Una muestra probabilística estratificada incluyó a 1.579 jóvenes de 12 a 17 años de 93 escuelas. Con el cuestionario se evaluó la violencia sexual en la familia y en la escuela. Se estimaron modelos mediante análisis de regresión logística, calculando la razón de momios *(odds ratio,* OR).

**Resultados.:**

El 18,68 % (IC_95%_: 16,80-20,60) sufrió algún tipo de agresión sexual. Además, el 9,75 % (IC_95%_: 8,28-11,21) informó haber sido tocado en alguna parte del cuerpo y el 1,84 % (IC_95%_: 1,17-2,50) informó que fue víctima de violación. La edad se identificó como factor de riesgo en el microsistema (OR=1,48) (IC_95%_: 1,26-1,74), mientras que la edad de la primera violencia sexual actuó como factor protector (OR=0,61) (IC_95%_: 0,54-0,69). Además, en el macrosistema, la percepción de que la violencia ocurre principalmente fuera del hogar incrementó el riesgo (OR=2,06) (IC_95%_: 1,01-4,19).

**Conclusión.:**

Aproximadamente, dos de cada diez encuestados informaron haber experimentado algún tipo de violencia sexual, siendo el acoso verbal y el contacto personal invasivo los más comunes. Ningún nivel del modelo ecológico o factor único puede explicar completamente la violencia sexual contra los adolescentes sin considerar su interconexión ecológica.

## Abstract (en)

**Introduction.:**

Sexual violence against adolescents is a global problem that affects young people around the world. The ecological model examines its forms and determinants through interconnected levels.

**Objective.:**

To determine the frequency, characteristics, and predictors of sexual violence in adolescents attending school in Perú.

**Materials and methods.:**

This was a cross-sectional study that secondarily analyzed data from the *Encuesta Nacional de Relaciones Sociales* (2019). A stratified probabilistic sample involved 1,579 youth aged 12-17 from 93 schools. The questionnaire evaluated physical, psychological, and sexual violence in the family and at school. Models were estimated using logistic regression analysis, calculating odds ratio (OR).

**Results.:**

Eighteen point sixty eight per cent (95% CI: 16.80-20.60) suffered some type of sexual assault. In addition, 9.75% (95% CI: 8.28-11.21) reported having been touched in some part of the body and 1.84% (95% CI: 1.17-2.50) reported that was a victim of rape. Age was identified as a risk factor in the microsystem (OR = 1.48) (95% CI: 1.26-1.74), while the age of the first experienced sexual violence acted as a protective factor (OR = 0.61) (95% CI: 0.54-0.69). In addition, in the macrosystem, the perception that violence occurs mainly outside the home increased the risk (OR = 2.06) (95% CI: 1.01-4.19).

**Conclusions.:**

Approximately two out of ten respondents reported having experienced some type of sexual violence, with verbal harassment and invasive personal contact being the most common. No single level or factor can fully explain adolescent sexual violence without considering its ecological interconnectedness.

La violencia sexual contra adolescentes es un problema social y de salud pública que los afecta gravemente en todo el mundo ^1^^,^^2^. De hecho, a nivel global, aproximadamente la mitad de los niños y adolescentes de 2 a 17 años experimentan anualmente algún tipo de violencia ^3^. Las consecuencias a corto y largo plazo de la violencia sexual en la adolescencia causan daño significativo a la salud física y mental de las personas afectadas a lo largo de su vida ^1^^,^^3^^,^^4^. Estos impactos abarcan infecciones de transmisión sexual y embarazos no deseados en etapas tempranas, así como trastornos como depresión y síntomas traumáticos ^5^^,^^6^.

El abuso perpetrado por personas cercanas al adolescente, como familiares de primer y segundo grado, está estrechamente vinculado a importantes síntomas de estrés 4,5. Otras consecuencias incluyen dificultades emocionales, comportamentales y sexuales, junto con sentimientos de vergüenza e inseguridad ^7^. Además, los afectados tienen mayores probabilidades de abandonar permanentemente la escuela, lo cual también implica pérdida de capital humano y el refuerzo de los ciclos de pobreza ^1^^,^^8^.

Siendo la adolescencia una etapa de búsqueda de identidad propia, independencia, creatividad y desarrollo interpersonal, muchas veces se encuentran expuestos a la violencia sexual en diversos ámbitos: familiar, escolar y comunitario, como ocurre en el Perú ^9^^-^^11^. Dentro del hogar, tanto familiares cercanos como conocidos de la familia son frecuentemente responsables de estos actos de violencia ^8^. Mientras que, en el ámbito escolar, también se presentan estas situaciones de acoso sexual entre compañeros ^6^^,^^9^^,^^10^. Estos actos pueden manifestarse mayoritariamente en forma de comentarios o bromas de carácter sexual, así como miradas hacia las partes íntimas, lo cual genera un ambiente hostil y contribuye a su vulnerabilidad ^12^. Además, se han reportado igualmente casos de violencia sexual en relaciones de pareja entre adolescentes ^5^^,^^10^.

Es fundamental reconocer que la violencia sexual contra los adolescentes no solo tiene lugar en el hogar y en la escuela, sino también, en su comunidad ^13^. No obstante, a pesar de que muchos adolescentes pueden reconocer y hacer valer sus derechos, los abusos suelen ocurrir en lugares aparentemente seguros, lo cual hace que los jóvenes se sientan desprotegidos y desconfiados en su entorno cotidiano ^5^^,^^8^. Además, las plataformas en línea también son escenarios de violencia sexual, es decir, el ámbito digital amplía la victimización en los menores ^2^^,^^3^. Esta serie de contextos diversos exige un enfoque integral en su abordaje ^13^^,^^14^.

De hecho, la Organización Mundial de la Salud (OMS) ^15^ propone el modelo ecológico de la violencia desarrollado por Lory Heise ^16^ como una herramienta conceptual que permite comprender la violencia de manera exhaustiva en sus diversas manifestaciones (psicológica, económica, física y sexual) en diferentes niveles interrelacionados. Estos sistemas abarcan factores personales, sociales y culturales, así como la interacción entre factores predictivos y protectores ^15^^,^^16^. De este modo, siguiendo la concepción de Heise sobre la exposición a la violencia en los ambientes sociales de historia personal, microsistema, exosistema y macrosistema, se analizan tanto los tipos de victimización por violencia sexual como los posibles factores predictivos ^14^^-^^16^.

En estudios peruanos previos, se señalaron prevalencias de violencia sexual en adolescentes entre el 28,5 (2016) ^9^ y el 25,6 % (2018) ^10^. No obstante estos datos susciten preocupación, aún resultan escasos los estudios nacionales sobre la prevalencia de violencia sexual y sus características en los adolescentes. Algunos de los acercamientos realizados, sin embargo, frecuentemente no permiten hacer inferencias a nivel nacional, o no siguen un modelo teórico que sustente el análisis de la interacción de factores protectores y de riesgo ^9^^,^^10^. Por tanto, es necesario realizar estudios para comprender mejor sus características y causas. Este estudio brinda estimaciones a nivel nacional, abordando la exposición de los jóvenes a la violencia sexual y sus factores determinantes en diferentes contextos, con el enfoque ecológico propuesto por Bronfenbrenner ^17^ y detallado por Heise ^16^.

Se empleó una encuesta nacional ^12^ en escuelas para investigar la prevalencia de la violencia sexual, las discrepancias de género en el autorreporte, las características de las formas de violencia y los posibles factores de riesgo.

La evaluación de la violencia sigue el enfoque conceptual de Mathews sobre abuso sexual en adolescentes ^18^, basándose en cuatro criterios:

- la persona que lo experimenta es un adolescente (según lo establecido legal y evolutivamente);
- la falta de consentimiento;
- el acto perpetrado es de naturaleza sexual (incluyendo relaciones sexuales, así como contacto físico abusivo, y abuso sin contacto físico (esto abarca comportamientos de acoso sexual que no implican contacto físico, como la difusión en línea de material íntimo o pornográfico, acoso sexual verbal o agresión), y
- dicho acto constituye abuso (en términos de relaciones de poder, desigualdad y explotación de la vulnerabilidad).

Con base en lo expuesto, el propósito fue determinar, mediante el modelo ecológico, la frecuencia, las características y los factores predictores de la violencia sexual en adolescentes escolarizados en Perú.

## Materiales y métodos

### Diseño

Se trata de un estudio de corte transversal, en el cual se analizaron secundariamente datos de la Encuesta Nacional de Relaciones Sociales ^12^, recolectados entre octubre y noviembre de 2019 por el Instituto Nacional de Estadística e Informática.

El estudio incluyó 1.579 jóvenes de 12 a 17 años provenientes de 93 escuelas secundarias (67 urbanas y 26 rurales) peruanas. Se utilizó un método de muestreo probabilístico, estratificado y de tres etapas: la unidad primaria era la institución educativa; la unidad secundaria, la sección seleccionada, y, finalmente, el adolescente representó la unidad terciaria. Asimismo, la cobertura de la encuesta implicó las 24 regiones peruanas y posee inferencia nacional.

### Variables y mediciones

El cuestionario electrónico referente a la violencia física, psicológica y sexual infligida a los adolescentes en el ámbito familiar y escolar fue dirigido por encuestadoras entrenadas y calificadas mediante la entrevista directa. Además, en el año 2013, el Instituto Nacional de Estadística e Informática evaluó las características psicométricas del mismo.

Respecto a la violencia sexual, se consideraron las respuestas de sí y no en 11 preguntas que figuraban en una tarjeta que se entregaba al encuestado y que abordaron, si alguna vez o en la actualidad, ocurría cualquiera de las siguientes situaciones en cualquier lugar de convivencia: "¿Te miran o te han mirado tus partes íntimas que te han hecho sentir mal o incómoda/o?, ¿Alguien te hace o te hizo comentarios o bromas de tipo sexual?, ¿Te obligan o te han obligado a ver pornografía en revistas, fotos, figuras o por internet?, ¿Alguien ha tratado o te ha quitado la ropa en contra de tu voluntad?, ¿Te obligan o te han obligado a realizar tocamientos o manoseos al cuerpo de otra persona?, ¿Eres o has sido víctima de tocamientos incómodos en alguna parte de tu cuerpo?, ¿Alguien se ha masturbado delante de ti?, ¿Alguien te obliga o te ha obligado a masturbarte?, ¿Alguien te muestra o te ha mostrado sus genitales?, ¿Te amenazan o has sido amenazada/o para tener relaciones sexuales?, ¿Te han obligado o te obligan a tener relaciones sexuales?" ^12^.

Mientras que, para estimar la prevalencia de la violencia sexual, se consideró una pregunta orientada a los últimos 12 meses: ¿Te ha ocurrido alguna de las situaciones mencionadas anteriormente?, clasificada como dicotómica. En este sentido, el Instituto Nacional de Estadística e Informática en conformidad con la ley peruana N° 30364 para esta variable, se refiere a cualquier acción sexual realizada sin consentimiento o bajo coerción. Incluye acciones que no requieren necesariamente penetración o contacto físico ^19^.

Se distribuyeron las variables en cuatro niveles de acuerdo con el modelo ecológico de Heise ^16^. De esta manera, la historia personal incluyó la edad (numérica discreta); el sexo y la edad en que experimentaron por primera vez violencia sexual (numérica discreta); idioma que prevalentemente se habla en casa (castellano, quechua, aimara, otra lengua nativa, idioma extranjero).

Por otro lado, en el microsistema se encuentran: miembros del hogar (madre, padre, hermana, hermano); frecuencia de peleas en el hogar (rara vez, algunas veces, casi siempre); persona que se encarga de su crianza trabaja (sí, no); se queda solo en casa (sí, no); momento en que se queda solo (mañana, la tarde, noche, todo el día); frecuencia de quedarse solo (todos los días, algún día laborable, sábados y domingo); falta al colegio para ayudar en casa (sí, no); solicitud de ayuda después de la violencia (sí, no); familiar al que se solicitó ayuda (madre, padre, hermana, hermano); recibió ayuda (sí, no); formas en las que recibió ayuda (consejos, charla con los familiares, confrontar el agresor, avisar a las autoridades).

En el exosistema se encuentra: región de residencia (costa, sierra, selva); área de residencia (urbana, rural); y, finalmente, en el macrosistema: frecuencia en considerar su opinión (rara vez, algunas veces, casi siempre o siempre); percepción sobre derechos en la adolescencia y creencias sobre la violencia sexual.

### Análisis estadístico

Previamente, se tuvieron en cuenta las complejas características muestrales de la encuesta, incluidos los pesos estratificados y de diseño. Para el análisis estadístico, se empleó el *software* Stata™, versión 15.1, estimando frecuencias, porcentajes, medidas de tendencia central y medidas de dispersión calculadas con base en la naturaleza de las variables.

Se utilizaron las pruebas de ji alcuadrado, Kruskal-Wallis y ANOVA para explorar asociaciones entre las variables. Después de haber obtenido resultados significativos en el análisis bivariado, se estimó inicialmente el factor de inflación de la varianza para detectar y corregir la multicolinealidad en el análisis de regresión. Se eliminaron las variables que presentaban un valor VIF *(Variance Inflation Factors)* mayor de 5, lo que mejoró el ajuste del modelo. Posteriormente, se hizo un análisis ajustado de regresión logística mediante las razones de probabilidad *(odds ratio,* OR).

Se establecieron cinco modelos, cuyas probabilidades indican el aumento o la disminución de la violencia sexual relacionada con un cambio unitario en los factores predictores o protectores. En el primer modelo, se evaluó la probabilidad de agresión sexual con los factores personales, mientras que, en el segundo modelo, se examinaron los efectos de factores microsistémicos. En el tercer modelo, se analizó la probabilidad de violencia sexual en relación con los factores exosistémicos y, en el cuarto modelo, se evaluaron los efectos de los factores macrosistémicos. Finalmente, en el quinto modelo, se incorporaron los cuatro niveles ecológicos para considerar el efecto de su interacción en la violencia sexual.

De cada modelo, se calculó el área bajo la curva y su ajuste se evaluó mediante el criterio de información de Akaike. Se calcularon los intervalos de confianza al nivel del 95 % y los valores de p por debajo de 0,05 se consideraron significativos.

### Aspectos éticos

Se analizó la base de datos pública obtenida del Instituto Nacional de Estadística e Informática. En primer lugar, es importante destacar que los datos de la encuesta recopilados se han anonimizado por completo, lo que garantiza que no se pueda identificar la identidad de los encuestados, salvaguardando su privacidad y confidencialidad. Además, el estudio cuenta con la aprobación del Comité de Ética Institucional de la Universidad Católica Sedes Sapientiae. Con la aprobación se han seguido rigurosamente todas las normas éticas en investigación.

## Resultados

En el cuadro 1 se detallan las formas de exposición a la violencia más frecuentes entre los adolescentes. El 7,22 % (IC_95%_: 5,90-8,50) reportó recibir miradas hacia sus partes íntimas que los hizo sentir incómodos, un 17,16 % (IC_95%_: 15,30-19,00) recibió comentarios o bromas de tipo sexual. Asimismo, el 9,75 % (IC_95%_: 8,28-11,21) reportó tocamientos en alguna parte de su cuerpo, el 1,84 % (IC_95%_: 1,17-2,50) sufrió o experimentaba alguna forma de abuso sexual. El 18,68 °% (IC_95%_: 16,80-20,60) de los adolescentes reportó, en los últimos 12 meses anteriores a la encuesta, alguna forma de violencia sexual, mientras que el 67 % (IC_95%_: 64,68-69,31) prefirió no responder.

Respecto a los factores individuales, la distribución de sexo fue similar (49,84 % mujeres y 50,16 % hombres), 14,47 años fue el promedio de edad y, 12,57 años, la media de inicio de la violencia sexual. En el microsistema, el 90,59 % vive en el hogar con la madre y el 70,25 % con el padre. Solamente un 7,8 % notificó a las autoridades sobre la violencia sexual. En el exosistema, el 84,17 % proviene de la zona urbana; en el macrosistema, el 54,80 % considera que la violencia sexual ocurre mayormente fuera de casa y, el 75,06 %, que ocurre más en sitios oscuros y solitarios (cuadro 1).

**Cuadro 1:** Descripción de la población de adolescentes, prevalencia y formas de violencia sexual con intervalos de confianza de 95 % (IC95%)

	n	% (IC95%)
¿Te miran o han mirado tus partes íntimas y te hacen sentir incómodo?		
Sí	114	7,22 (5,90-8,50)
No	1465	92,78 (91,50-94,05)
Comentarios o bromas de tipo sexual		
Sí	271	17,16 (15,30-19,00)
No	1308	82,84 (80,98-84,69)
¿Te obligan o te han obligado a ver pornografía?		
Sí	73	4,62 (3,58-5,65)
No	1506	95,38 (94,34-96,41)
¿Alguien ha tratado o te ha quitado la ropa en contra de tu voluntad?		
Sí	51	3,23 (2,35-4,10)
No	1528	96,77 (95,89-97,64)
¿Te obligan o te han obligado a realizar tocamientos al cuerpo de otra persona?		
Sí	50	3,17 (2,30-4,03)
No	1529	96,83 (95,96-97,69)
¿Has experimentado tocamientos incómodos en alguna parte de tu cuerpo'		
Sí	154	9,75 (8,28-11,21)
No	1425	90,25 (95,89-97,64)
¿Alguien se ha masturbado delante de ti?		
Sí	44	2,79 (1,97-3,60)
No	1535	97,21 (96,39-98,02)
¿Alguien te obliga o te ha obligado a masturbarte?		
Sí	10	0,63 (0,2-1,02)
No	1569	99,37 (98,97-99,76)
¿Alguien te muestra o te ha mostrado sus genitales?		
Sí	53	3,36 (2,47-4,28)
No	1526	96,64 (95,75-97,52)
¿Te amenazan o has sido amenazada/o para tener relaciones sexuales?		
Sí	27	1,71 (1,07-2,34)
No	1552	98,29 (97,65-98,92)
¿Te han obligado o te obligan a tener relaciones sexuales?		
Sí	29	1,84 (1,17-2,50)
No	1550	98,16 (97,49-98,82)
Violencia sexual en los últimos 12 meses		
Sí	295	18,68 (16,80-20,60)
No	226	14,31 (12,58-16,03)
No responde	1058	67,00 (64,68-69,31)
Factores individuales		
Sexo	787	49,84 (47,37-52,30)
Mujer	792	50,16 (47,69-52,62)
Hombre		
Edad		
media ± DE	14,47±1,45	(14,40-14,54)
Edad de la primera violencia sexual		
media ± DE	12,57±2,39	(12,45-12,69)
Idioma		
Castellano	1451	92,30 (90,98-93,61)
Quechua	93	5,92 (4,75-7,08)
Aimara	8	0,51 (0,15-0,86)
Otra lengua nativa	17	1,08 (0,57-1,58)
Idioma extranjero	3	0,19 (0,01-0,40)
Factores del microsistema		
Presencia de la madre		
Sí	1425	90,59 (89,14-92,03)
No	148	9,41 (7,96-10,85)
Presencia del padre		
Sí	1105	70,25 (67,99-72,50)
No	468	29,75 (27,49-32,00)
La persona que se encarga de su crianza trabaja		
Sí	882	56,29 (53,84-58,73)
No	685	43,71 (41,26-46,15)
¿Se queda solo en casa?		
Sí	674	42,85 (40,40-45,29)
No	899	57,15 (54,70-59,59)
El momento en que se queda sola/o es		
Toda la mañana	200	29,67 (27,41-31,92)
Toda la tarde	389	57,72 (55,28-60,15)
Toda la noche	25	3,71 (2,77-4,64)
Todo el día	60	8,90 (7,49-10,30)
Frecuencia de quedarse sola/o		
Todos los días	40	6,48 (5,26-7,69)
Algún día de lunes a viernes	323	52,35 (49,88-54,81)
Algún día de sábado a domingo	254	41,17 (38,74-43,59)
¿Falta al colegio para ayudar en la casa u otro lugar?		
Sí	109	6,93 (5,67-8,18)
No	1464	93,07 (91,81-94,32)
Frecuencia de peleas entre los padres		
Rara vez	461	48,22 (45,75-50,68)
Algunas veces	419	43,83 (41,38-46,27)
Siempre/Casi siempre	76	7,95 (6,61-9,28)
Persona a la que solicitó ayuda		
Madre		
Sí	105	48,61 (46,14-51,07)
No	111	51,39 (48,92-53,85)
Padre		
Sí	44	20,37 (18,38-22,35)
No	172	79,63 (77,64-81,61)
Profesora		
Sí	15	6,94 (5,68-8,19)
No	201	93,06 (91,80-94,31)
Profesor		
Sí	17	7,87 (6,54-9,19)
No	199	92,13 (90,80-93,45)
Recibió ayuda		
Sí	205	94,91 (93,82-95,99)
No	11	5,09 (4,00-61,74)
Formas de ayuda		
Confrontación al agresor		
Sí	77	37,56 (35,17-39,94)
No	128	62,44 (59,99-64,89)
Avisaron a las autoridades		
Sí	16	7,80 (6,47-9,12)
No	189	92,20 (90,87-93,52)
Acudir a un especialista		
Sí	19	9,27 (7,83-10,70)
No	186	90,73 (89,29-92,16)
Acudir a una institución para buscar ayuda		
Sí	23	4,41 (3,39-5,42)
No	498	95,59 (94,57-96,60)
Factores del exosistema		
Región		
Costa	679	43,00 (40,55-45,44)
Sierra	600	38,00 (35,60-40,39)
Selva	300	19,00 (17,06-20,93)
Área de residencia		
Urbana	1329	84,17 (82,36-85,97)
Rural	250	15,83 (14,02-17,63)
Factores del macrosistema		
Frecuencia en considerar su opinión		
Rara vez	139	9,79 (8,32-11,25)
Algunas veces	563	39,65 (37,23-42,06)
Siempre/Casi siempre	718	50,56 (48,09-53,02)
Percepción sobre los derechos		
Un adolescente debe trabajar cuando falta plata en casa		
Sí	458	29,28 (27,03-31,52)
No	1106	70,72 (68,47-72,96)
Un adolescente puede expresar lo que piensa y siente		
Sí	1515	96,50 (95,59-97,40)
No	55	3,50 (2,59-4,40)
Los padres pueden decidir que su hijo deje de estudiar		
Sí	213	13,67 (11,97-15,36)
No	1345	86,33 (84,63-88,02)
Los profesores tienen derecho de golpear para corregir		
Sí	69	4,40 (3,38-5,41)
No	1498	95,60 (94,58-96,61)
Los padres tienen derecho de golpear cuando su hijo se porta mal		
Sí	628	40,26 (37,63-42,46)
No	932	59,74 (57,32-62,15)
Creencias sobre la violencia sexual		
Solo es cometida por personas "locas"		
Sí	626	40,05 (37,63-42,46)
No	937	59,95 (57,53-62,36)
Un adolescente puede denunciar		
Sí	1543	97,91 (97,20-98,61)
No	33	2,09 (1,38-2,79)
Solo les ocurre a los menores pobres		
Sí	105	6,71 (5,47-7,94)
No	1460	93,29 (92,05-94,52)
Ocurre mayormente fuera de casa		
Sí	851	54,80 (52,34-57,25)
No	702	45,20 (42,74-47,65)
Ocurre más en sitios oscuros y solitarios		
Sí	1171	75,06 (72,92-77,19)
No	389	24,94 (22,80-27,07)

DE: desviación estándar

La violencia sexual se relacionó con la edad reportada del primer episodio de violencia (p = 0,000); en el grupo que reportó formas de violencia, la media de edad (m = 14,49) fue ligeramente inferior (cuadro 2). Respecto al microsistema, ante la presencia de violencia, los adolescentes suelen solicitar ayuda a la profesora (p = 0,020) y al profesor (p = 0,007) con mayor frecuencia. Asimismo, recibieron como formas de ayuda acudir a las autoridades (p = 0,032), a un especialista (p = 0,000) o a una institución (p= 0,010). En el exosistema, la violencia sexual se asoció con el área de residencia (p = 0,018) y se encontró que la mayoría de quienes reportan provienen del área urbana. Finalmente, en el macrosistema, los adolescentes que perciben que ocurre mayormente fuera de casa lo experimentaron en mayor proporción (p = 0,011). Por otro lado, es preciso señalar que no se encontró asociación con el sexo, dado que la violencia sexual se presentó en similar proporción en mujeres (55,0 %) y hombres (58,82 %) (cuadro 2).

**Cuadro 2:** Asociación entre la violencia sexual en adolescentes y los factores del modelo ecológico

	Violencia sexual en los últimos doce meses				
	Sí		No		p
	n	%	n	%	
Factores individuales					
Sexo					0,384
Mujer	165	55,00	135	45,00	
Hombre	130	58,82	91	41,18	
Edad					0,1778
media ± DE	14,49	1,40	14,64	1,41	
Edad de la primera violencia sexual					0,0000
media ± DE	13,31	1,88	11,62	2,64	
Idioma					0,740
Castellano	268	56,90	203	43,10	
Quechua	17	54,84	14	45,16	
Aimara	1	50,00	1	50,00	
Otra lengua nativa	4	40,00	6	60,00	
Idioma extranjero	1	100,00	0	0,00	
actores del microsistema					
Presencia de la madre					0,107
Sí	25	46,30	29	53,70	
No	267	57,79	195	42,21	
Presencia del padre					0,924
Sí	94	56,29	73	43,71	
No	198	56,73	151	43,27	
La persona que se encarga de su crianza trabaja					0,927
Sí	160	56,74	122	43,26	
No	129	56,33	100	43,67	
Se queda solo en casa					0,300
Sí	149	58,89	104	41,11	
No	143	54,37	120	45,63	
El momento en que se queda sola/o es:					0,084
Toda la mañana	34	47,22	38	52,78	
Toda la tarde	93	65,03	50	34,97	
Toda la noche	5	50,00	5	50,00	
Todo el día	17	60,71	11	39,29	
Frecuencia de quedarse sola/o					0,636
Todos los días	14	70,00	35,00	30,00	
Algún día de lunes a viernes	73	59,35	50	40,65	
Algún día de sábado a domingo	50	58,82	35	41,18	
Falta al colegio para ayudar en la casa u otro lugar					0,768
Sí	31	58,49	22	41,51	
No	261	56,37	202	43,63	
Frecuencia de peleas entre los padres					0,111
Rara vez	98	58,68	69	41,32	
Algunas veces	98	62,03	60	37,97	
Siempre/Casi siempre	21	53,85	18	46,15	
Persona a la que solicitó ayuda					
Madre					0,223
Sí	61	54,95	50	45,05	
No	49	46,67	56	53,33	
Padre					0,250
Sí	91	52,91	81	47,09	
No	19	43,18	25	56,82	
Profesora					0,020
Sí	98	48,76	103	51,24	
No	12	80,00	3	20,00	
Profesor					0,007
Sí	96	48,24	103	51,76	
No	14	82,35	3	17,65	
Amiga					0,497
Sí	100	50,25	99	49,75	
No	10	58,82	7	41,18	
Amigo					0,367
Sí	99	50,00	99	50,00	
No	11	61,11	7	38,89	
Recibió ayuda					0,805
Sí	104	50,73	101	49,27	
No	6	54,55	5	45,45	
Formas de ayuda					
Confrontación al agresor					0,154
Sí	60	46,88	68	53,13	
No	44	57,14	33	42,86	
Avisaron a las autoridades					0,032
Sí	100	52,91	89	47,09	
No	4	25,00	12	75,00	
Acudir a un especialista					0,000
Sí	102	54,84	84	45,16	
No	2	10,53	17	89,47	
Acudir a una institución para buscar ayuda					0,010
Sí	7	30,43	16	69,57	
No	288	57,83	210	42,17	
Factores del exosistema					
Región					0,060
Costa	119	57,49	88	42,51	
Sierra	110	61,80	68	38,20	
Selva	66	48,53	70	51,47	
Área de residencia					0,018
Urbana	264	58,67	186	41,33	
Rural	31	43,66	40	56,34	
Factores del macrosistema					
Frecuencia en considerar su opinión					0,066
Rara vez	24	44,44	30	55,56	
Algunas veces	108	55,38	87	44,62	
Siempre/Casi siempre	118	61,78	73	38,22	
Percepción sobre los derechos					
Un adolescente debe trabajar cuando falta plata en casa					0,542
Sí	100	58,48	71	41,52	
No	192	55,65	153	44,35	
Un adolescente puede expresar lo que piensa y siente					0,175
Sí	281	56,09	220	43,91	
No	13	72,22	5	27,78	
Los padres pueden decidir que su hijo deje de estudiar					0,003
Sí	53	72,60	20	27,40	
No	240	54,30	202	45,70	
Los profesores tienen derecho de golpear para corregir					0,922
Sí	19	57,58	14	42,42	
No	275	56,70	210	43,30	
Los padres tienen derecho de golpear cuando su hijo se porta mal					0,492
Sí	139	58,40	99	41,60	
No	154	55,40	124	44,60	
Creencias sobre la violencia sexual					
Solo es cometida por personas "locas"					0,266
Sí	117	60,00	78	40,00	
No	176	55,00	144	45,00	
Un adolescente puede denunciar					0,882
Sí	289	56,78	220	43,22	
No	6	54,55	5	45,45	
Solo les ocurre a los menores pobres					0,494
Sí	20	62,50	12	37,50	
No	272	56,31	211	43,69	
Ocurre mayormente fuera de casa					0,011
Sí	176	61,54	110	38,46	
No	117	50,43	115	49,57	
Ocurre más en sitios oscuros y solitarios					0,261
Sí	228	58,02	165	41,98	
No	67	41,98	61	47,66	

En el cuadro 3 se detallan los predictores según el modelo ecológico. En el modelo 1, los adolescentes con mayor edad se encuentran expuestos a la violencia sexual 1,48 veces más en comparación con los menores (OR = 1,48) (IC_95%_:1,26-1,74) (p < 0,000). Mientras que, una menor edad de reporte de la primera violencia sexual sufrida representa un factor de protección en los adolescentes (OR = 0,61) (IC_95%_: 0,54-0,69) (p < 0,000). Al igual que en el modelo 2, pedir ayuda al profesor es un factor protector ante un evento de violencia (OR = 0,26) (IC_95%_: 0,07-0,95) (p = 0,042). En el modelo 3, proceder de una zona urbana expone 1,81 veces más a la violencia sexual, en comparación con el área rural (OR = 1,81) (IC_95%_: 1,07-3,08) (p = 0,028). En el nivel 4, no percibir como derecho que los padres pueden decidir que su hijo deje de estudiar, tiene mayor exposición en adolescentes en comparación con los que sí están de acuerdo (OR = 2,14) (IC_95%_: 1,15-3,97) (p = 0,016), mientras que los adolescentes que no perciben que la violencia sexual ocurre mayormente fuera de casa, se exponen 1,69 más veces a esta, en comparación con los que sí lo perciben (OR = 1,69) (IC_95%_: 1,09-2,60) (p = 0,018). Finalmente, en el modelo 5 donde interactúan los cuatro niveles considerados del enfoque ecológico, la edad de los adolescentes y la edad reportada de la primera violencia sexual sufrida, incrementaron sus valores de exposición (OR = 1,59) (IC_95%_: 1,21-2,09) (p < 0,000) y los de protección (OR = 0,68) (IC 95%: 0,58-0,81) (p < 0,000). Asimismo, los adolescentes que no perciben que la violencia sexual ocurre mayormente fuera de casa, se han expuesto 2,06 veces más a esta, en comparación con los que sí perciben que ocurre fuera de casa.

**Cuadro 3:** Predictores de la violencia sexual en adolescentes según el modelo ecológico

	Modelo 1			Modelo 2			Modelo 3			Modelo 4			Modelo 5		
	OR	IC95%	p	OR	IC95%	p	OR	IC95%	p	OR	IC95%	p	OR	IC95%	p
Factores individuales															
Sexo															
Mujer	1,0	-	-										1,0	-	-
Hombre	1,09	0,74-1,62	0,659										0,84	0,42-1,69	0,621
Edad	1,48	1,26-1,74	0,000										1,59	1,21-2,09	0,001
Edad de la primera violencia sexual	0,61	0,54-0,69	0,000										0,68	0,58-0,81	0,000
Idioma															
Castellano	1,0	-	-												
Quechua	1,47	0,66-3,24	0,346												
Aimara	0,81	0,04-16,65	0,887												
Otra lengua nativa	2,29	0,58-9,01	0,234												
Factores del microsistema															
La persona que se encarga de su crianza trabaja															
Sí				1,0	-	-							1,0	-	-
No				0,82	0,46-1,46	0,501							0,83	0,44-1,58	0,575
¿Se queda solo en casa?															
Sí				1,0	-	-							1,0	-	-
No				0,83	0,47-1,47	0,520							0,81	0,43-1,52	0,511
Persona a la que solicitó ayuda															
Padre															
Sí				1,0	-	-									
No				1,48	0,74-2,97	0,270									
Hermana															
Sí				1,0	-	-							1,0	-	-
No				0,55	0,17-1,78	0,318							0,50	0,14-1,80	0,285
Profesor															
Sí				0,26	0,07-0,95	0,042							0,28	0,07-1,18	0,083
No				1,0	-	-							1,0	-	-
Formas de ayuda															
Confrontación al agresor															
Sí				1,0	-	-									
No				0,68	0,38-1,24	0,212									
Avisaron a las autoridades															
Sí															
No															
Acudir a un especialista															
Sí															
No															
Factores del exosistema															
Región															
Costa							1,0	-	-						
Sierra							0,75	0,49-1,15	0,189						
Selva							1,25	0,80-1,97	0,327						
Área de residencia															
Urbana							1,0	-	-						
Rural							1,81	1,07-3,08	0,028						
**Factores del macrosistema**															
Frecuencia en considerar su opinión															
Rara vez										1,0	-	-			
Algunas veces										0,70	0,37-1,33	0,282			
Siempre/Casi siempre										0,56	0,29-1,06	0,075			
Percepción sobre los derechos															
Un adolescente debe trabajar cuando falta plata en casa															
Sí										1,0	-	-			
No										1,08	0,69-1,70	0,723			
Un adolescente puede expresar lo que piensa y siente															
Sí										1,0	-	-			
No										0,33	0,07-1,60	0,167			
Los padres pueden decidir que su hijo deje de estudiar															
Sí										1,0	-	-			
No										2,14	1,15-3,97	0,016			
Los profesores tienen derecho de golpear para corregir															
Sí										1,0	-	-			
No										0,82	- 0,37-1,85	0,635			
Los padres tienen derecho de golpear cuando su hijo se porta mal															
Sí										1,0	-	-	1,0	-	-
No										1,10	0,72-1,67	0,656	0,63	0,33-1,22	0,170
Percepción sobre la violencia sexual Solo es cometida por personas “locas”															
Sí										1,0	-	-			
No										1,23	0,80-1,90	0,345			
Un adolescente puede denunciar															
Sí										1,0	-	-			
No										2,10	0,47-9,39	0,334			
Solo les ocurre a los menores pobres															
Sí										1,0	-	-			
No										0,82	0,36-1,87	0,642			
Ocurre mayormente fuera de casa															
Sí										1,0	-	-	1,0	-	-
No										1,69	1,09-2,60	0,018	2,06	1,01-4,19	0,046
Ocurre más en sitios oscuros y solitarios															
Sí										1,0	-	-	1,0	-	-
No										1,02	0,61-1,69	0,948	0,57	0,26-1,25	0,164

OR: *odds ratio*; IC_95%_: intervalo de confianza del 95 %

**Cuadro 4:** Comparación de parámetros de los cinco modelos de predicción de la violencia sexual en adolescentes peruanos

	Sensibilidad	Especificidad	Clasificación	Hosmer- Lemeshow	AIC	AUC
	(%)	(%)	(%)			
Modelo 1	46,88	88,28	70,23	0,0000	627,07	0,7807
Modelo 2	75,00	57,69	66,96	0,2960	157,57	0,7253
Modelo 3	10,62	95,25	58,54	0,1862	710,60	0,5756
Modelo 4	40,68	79,17	62,83	0,5290	571,83	0,6332
Modelo 5	61,46	76,24	69,04	0,1122	256,11	0,7523

AIC: *Akaike information criteria;* AUC: *Area under the curve*

**Figura 1:**
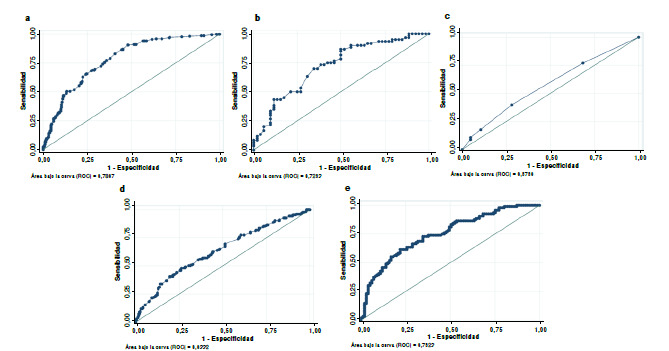
Representación gráfica de las curvas ROC (característica operativa del receptor) y el área bajo la curva de los modelos de predicción de violencia sexual. a) Modelo 1: factores de la historia personal, AUC = 0,78; b) Modelo 2: factores del microsistema, AUC = 0,72; c) Modelo 3: factores del exosistema, AUC = 0,57; d) Modelo 4: factores del macrosistema, AUC = 0,63; e) Modelo 5: combinación de los demás modelos, AUC = 0,75

Con posterioridad, se estimó la capacidad discriminante de cada modelo con el área bajo la curva. En el cuadro 4 se refleja la comparación de los parámetros de predicción de cada uno de estos. Mientras que, en la figura 1, se representan la curva característica del receptor para cada modelo y sus respectivas AUC. Para el modelo 1, el AUC fue de 0,78 y, para el modelo 2, de 0,72. Por su parte, en el modelo 5, el área bajo la curva fue de 0,75 con los valores menores de AIC (256,11).

## Discusión

En el modelo ecológico, la violencia sexual en adolescentes se comprende a través de la interacción de múltiples factores que coexisten en niveles interconectados. Es así como esta interacción puede influir en la magnitud predictiva de los factores asociados con la violencia 15,16. Por lo tanto, se propuso como objetivo determinar la frecuencia, las características y los factores predictores de la violencia sexual en adolescentes escolarizados en Perú.

Nuestros hallazgos señalan que casi uno de cada cinco adolescentes encuestados ha sufrido algún tipo de violencia sexual en el último año, y alrededor del 2 % ha sido forzado a tener relaciones sexuales. Otros estudios en Latinoamérica muestran igualmente una gran prevalência de violencia sexual entre los adolescentes de 12 a 17 años ^2^^,^^9^. Sin embargo, la violencia sexual no es un problema exclusivo de esta región y varía según el contexto sociocultural de cada país o zona ^4^^,^^20^. Además, la prevalencia identificada de violencia sexual entre los encuestados refleja una cultura machista y patriarcal que tiende a la cosificación y objetivación del cuerpo ^2^^,^^4^. Si esta se produce de manera constante y duradera dentro de las familias, contribuye a que se acepte y se perpetúe el ciclo de violencia en las futuras generaciones ^1^^,^^2^^,^^5^. En este sentido, el modelo ecológico reconoce que las normas culturales que legitiman la violencia, los valores patriarcales y las disparidades económicas y legales, son factores que influyen en las tasas de violencia sexual ^14^^,^^21^. Por ello, se requiere de servicios de apoyo y programas de prevención adecuados a cada realidad ^2^^,^^20^.

Partiendo de los factores individuales del enfoque ecológico, se destaca que el reporte de la violencia sexual fue similar independientemente del sexo. Esta similitud podría deberse a que las normas sociales discriminatorias y el abuso de poder de los adultos afectan tanto a mujeres como a varones en el Perú. La mayoría de los agresores sexuales son hombres adultos que gozan de impunidad y rara vez son castigados ^21^^,^^22^. Sin embargo, aún queda mucho por esclarecer por medio de una mayor desagregación de los datos por género.

Se apreció que las formas de violencia más frecuentes fueron los comentarios de tipo sexual, los tocamientos y las miradas incómodas. Estos comportamientos necesitan una mayor profundización a fin de comprender mejor las diferencias respecto al sexo de quienes los experimentan. El acoso visual o verbal y el contacto o acoso personalmente invasivo son los más reportados por la literatura en adolescentes ^22^^-^^24^. De hecho, la violencia sexual que involucra contacto e invasión del espacio personal, generalmente, se considera más grave y suele ir acompañada de comportamientos visuales o verbales ^9^^,^^22^. Esto indica, por un lado, que estas formas de violencia tienen una jerarquía y, por el otro, que los adolescentes que sufren comportamientos de contacto o invasión personal podrían experimentar más formas de violencia sexual ^7^^,^^22^^,^^24^.

Se apreció que los adolescentes con mayor edad tienen un 48 % más de riesgo de estar expuestos a la violencia sexual que los menores. La edad es una dimensión crucial para comprender la violencia sexual ^25^^,^^26^, ya que los niños, niñas y adolescentes tienen derechos legales, poder social, económico y político limitados ^14^^,^^26^. Esto los hace vulnerables a la agresión y explotación de los adultos que carecen de estas limitaciones ^2^^,^^14^. Una posible explicación de este incremento en el riesgo es que los adolescentes de más edad han vivido más circunstancias, como encuentros sexuales, y pueden tener una mayor exposición a alguna forma de abuso ^25^. Además, en esta etapa suelen tener más curiosidad y actividad sexual, así como influencias sociales de sus pares, lo que también aumentaría la posibilidad de encuentros sexuales no deseados ^21^^,^^25^.

Por otra parte, en el microsistema, buscar la ayuda de un profesor protegió a los adolescentes de la violencia sexual en su entorno inmediato. Por ejemplo, de comentarios sexuales, bromas, gestos o miradas ^27^. Los docentes educan a los adolescentes para desarrollar su autoconciencia, regulación y habilidades sociales ^28^. En efecto, estas últimas favorecen el desarrollo de la autoestima, la confianza y el apoyo mutuo, ayudando a la prevención de conductas agresivas y coercitivas ^28^^,^^29^. En este sentido, mediante la creación de aulas seguras, los docentes promueven las relaciones respetuosas entre pares, abordan la violencia, conectan a los estudiantes con servicios de apoyo y mejoran las estrategias escolares de prevención ^27^^,^^29^.

Según nuestros resultados, solo el 7,8 % de los afectados acudió a las autoridades, mientras que el resto recurrió a familiares, amigos, profesores, especialistas e instituciones. La situación es grave, pero muchos adolescentes, incluyendo varones, no reportan la violencia sexual ^5^. En este sentido, otro elemento revelador de nuestros hallazgos es la gran tasa de abstención a la pregunta de prevalencia de violencia en los últimos doce meses. Una posible explicación es que, con frecuencia, individuos cercanos y de confianza para el adolescente cometen estos actos abusivos ^5^^,^^7^. En los casos en que el abusador es un miembro de la familia, un amigo, una figura de autoridad o alguien en una posición de poder, el adolescente puede sentirse impotente para hablar debido a preocupaciones sobre represalias o consecuencias negativas ^5^^-^^7^. Además, varios elementos, como el temor a las repercusiones, la vergüenza, la culpa, el miedo a no ser creído o afrontar juicios, y el desconocimiento de los derechos propios y de los recursos disponibles para recibir apoyo y protección, pueden afectar la notificación de las agresiones sexuales ^30^^,^^31^. Otra explicación para el escaso reporte a las autoridades es que las personas que vivencian violencia sexual pueden experimentar diversas emociones que les generan confusión, lo cual dificulta la comprensión sobre lo ocurrido y expresarlo ^30^^,^^31^.

En el exosistema, los adolescentes que viven en áreas rurales tienen un 81 % más de riesgo de experimentar violencia sexual que los que viven en áreas urbanas. Esta situación coincide con estudios de países africanos ^25^ y de Latinoamérica ^1^^,^^2^^,^^6^. Como señala el enfoque ecológico, la interacción entre los niveles puede aumentar el riesgo de violencia sexual ^16^. En este sentido, vivir en una zona rural se relaciona con otros factores potenciales, como las circunstancias familiares y de vida, que incluyen un nivel socioeconómico más bajo y niveles de educación familiar inferiores ^6^. Estos elementos interactúan y se relacionan con otras vulnerabilidades económicas de los jóvenes, así como vivir en una familia no nuclear, los cuales se asocian con la violencia sexual ^6^^,^^32^.

Otro posible elemento explicativo del riesgo asociado con el lugar de residencia se vincula al apoyo de la comunidad. En algunos lugares, la comunidad puede ser más solidaria con las personas afectadas por la violencia sexual. Donde el contexto comunitario es más fuerte, esto facilitaría su recuperación y empoderamiento ^6^^,^^21^. Por lo cual, los profesionales que no aplican protocolos basados en las características sociales, culturales y económicas de la comunidad, más aún en poblaciones rurales, pueden equivocarse ignorando o minimizando los indicadores de violencia sexual ^13^.

Por otro lado, las creencias y los valores culturales pertenecientes al macrosistema reflejan que la falta de percepción de la violencia sexual fuera del hogar aumenta el riesgo de sufrirla. No obstante, esta ocurre principalmente en el hogar, según estudios en Brasil ^33^ y Colombia ^34^, donde los agresores son principalmente hombres jóvenes y familiares de distintos grados, siendo los padres los más frecuentes. Sin embargo, es importante señalar que el riesgo de sufrir violencia sexual no está directamente relacionado solo con la percepción, sino que también está influenciado por diversos factores sociales, culturales e individuales ^20^^,^^21^. En este sentido, la falta de medidas de prevención y educación en la sociedad puede repercutir en la falta de percepción o conciencia de la violencia sexual entre los encuestados. Los adolescentes que no son conscientes de la posibilidad de sufrir violencia sexual dentro o fuera del hogar, probablemente no tomen precauciones adecuadas para protegerse.

Respecto a las creencias adolescentes acerca de la violencia sexual, como las ideas erróneas sobre la violación ^35^ y la empatía hacia las víctimas o los agresores ^36^, abundan en la investigación. De hecho, el modelo ecológico señala que estas creencias permean y dan forma a los otros niveles ecológicos ^16^. A nivel individual y comunitario, las creencias que exculpan el uso de la violencia y las normas sociales de género han sido exploradas como importantes factores de riesgo ^1^^,^^2^. Sin embargo, no hay estudios previos sobre la relación entre las creencias sobre el lugar de la violencia sexual y la probabilidad de experimentarla, por lo que es necesario investigar más en esta área. Simultáneamente, otro aspecto que limita su explicación es el diseño transversal empleado, el cual impide interpretar causalmente estas creencias y la violencia sexual.

Finalmente, el efecto predictivo y de protección a nivel individual fue potenciado por el modelo combinado. Además, los factores en el microsistema y el exosistema no modifican su efecto lo que indica cierta autonomía de otros niveles, mientras que, los factores del macrosistema incrementaron su efecto de riesgo. De esta manera, la acción concurrente de los niveles y su anidación, varían en términos de su poder predictivo a través de los niveles ecológicos ^6^^,^^32^. Por lo tanto, al incorporar todos estos factores, el modelo 5 demostró la mayor capacidad discriminatoria, apoyando así la tesis de considerar la ecología integral del individuo ^16^^,^^17^.

El estudio tiene algunas limitaciones, como el uso de autorreportes y la posibilidad de subreporte, que pueden afectar las proporciones de violencia sexual identificadas. Sin embargo, la OMS valora las encuestas nacionales y el muestreo por conglomerados para obtener conocimiento a nivel nacional. A pesar de estas limitaciones, el estudio se basa en una muestra de adolescentes escolarizados representativa a nivel nacional, lo que permite identificar características específicas de los afectados por violencia sexual, con un riguroso análisis estadístico y un marco conceptual aplicado. Además, el estudio indaga a los adolescentes sobre sus experiencias de la infancia y de los últimos doce meses, en lugar de basarse en informes retrospectivos de violencia sexual infantil de adultos, lo que minimiza el sesgo de memoria.

En conclusión, la violencia sexual es un problema de salud pública que afecta a casi una quinta parte de los adolescentes peruanos escolarizados y se relaciona con diversos factores personales, familiares, sociales y culturales que pueden aumentar o disminuir su riesgo. Además, el estudio muestra que la violencia sexual contra los adolescentes no se puede explicar solo por los factores de riesgo o de protección de cada nivel ecológico, sino por la interacción entre ellas. Sin embargo, esta interacción no es igual para todos los niveles y factores, ya que algunos tienen más capacidad predictiva que otros.

En este sentido, las políticas públicas deben abordar los factores de riesgo de la violencia sexual en todos los niveles, desde lo individual hasta lo sociocultural. Sin embargo, en este estudio se destaca que las actividades de prevención primaria deben enfocarse en el microsistema. El papel de los educadores es clave para desarrollar habilidades sociales en los adolescentes, que les permitan protegerse y prevenir la violencia sexual.
